# Cybersecurity threats and vulnerabilities experienced by small-scale African migrant traders in Southern Africa

**DOI:** 10.1057/s41284-023-00378-1

**Published:** 2023-06-10

**Authors:** Paul Kariuki, Lizzy Oluwatoyin Ofusori, Prabhakar Rontala Subramaniam

**Affiliations:** School of Management, IT and Governance, University of KwaZulu-Natal, Durban, South Africa

**Keywords:** Cybersecurity, Threats and vulnerabilities, Small-scale traders

## Abstract

Cybersecurity threats have increased as the world becomes increasingly interconnected. Whilst the use of technology to facilitate commercial activities is now common practice, there is a need to limit exposure to these threats so that traders can transact safely. This study aimed to identify and analyse common cybersecurity vulnerabilities and threats experienced by small-scale African migrant traders in Southern Africa. A qualitative approach was employed and semi-structured and key informant interviews were conducted to gather the primary data, with secondary data sourced from the relevant literature. The study found that the majority of the small-scale traders experienced hacking while using their mobile devices for transacting. Moreover, most reported a lack of knowledge of cybersecurity and were therefore vulnerable to further threats. It is recommended that small-scale traders be capacitated with relevant technical information to enhance their understanding of cybersecurity risks that can negatively affect their commercial activities. There is also a need for further research to identify mitigation techniques and infrastructure to protect small-scale traders.

## Introduction

The use of cyber technologies has increased in today’s globalised world, transforming society, business, and economies in unprecedented ways. However, exponential growth in the use of cyberspace has resulted in an increase in cybercriminal activities (Monteith et al. 2021). The main reason is excessive use of web-based applications, given the reality of the COVID-19 pandemic that has increased online interaction (Buil-Gil et al. 2021). Cybercriminals use web applications to obtain confidential information and disrupt economic transactions (Palmieri et al. 2021) by businesses, governments, institutions and ordinary individuals (Chigada and Madzinga 2021). It is against this background that research on cybersecurity has gained traction among academics and practitioners.

For the purposes of this study, cybersecurity is protection against unauthorised use of electronic means to attack systems and disrupt activities. Conteh (2021) stated that cybersecurity is the practice of defending electronic equipment such as computers, servers, mobile devices and electronic systems and data from malicious attacks. It is evident that cybersecurity is relevant in a variety of contexts from business to mobile computing and information systems and networks, all of which are susceptible to cyberattacks (Dupont and Whelan 2021). It is imperative that cybersecurity systems be enhanced so that malicious attacks can be detected, prevented, and timeously responded to, as a range of cyber threats are arising from variety of context (Horgan et al. 2021).

Cybercrime refers to offensive attacks that target the information systems of servers, computers, computer networks, and infrastructure, including personal computers and mobile devices (Kamiya et al. 2021; Minnaar 2020). The primary objective is to disable systems, steal information and render the entire system inefficient (Lallie et al. 2021). The methods used in cyberattacks include malware, phishing, and ransomware, among others (ur Rehman et al. 2021). This calls for ongoing research to develop security systems that can address different breaches and mitigate them through early detection and prevention.

While small-scale businesses experience the same cybersecurity threats as large enterprises, small-scale traders subscribe to the misconception that they are too small and obscure to be targeted (Banham 2017). However, this is not the case as increasing automation enables cybercriminals to target hundreds, if not thousands, of small-scale businesses simultaneously (Joel 2021). According to Fuentes (2020), small businesses often have less stringent technological defences, are less aware of threats and have less time and resources to invest in cybersecurity. This makes them an easier target for hackers than bigger organisations.

This study examined how cybersecurity threats and vulnerabilities impact small-scale African migrant traders in Southern Africa. Small-scale African migrant traders are defined as entrepreneurs whose business activities are small enough not to be regulated and that have few employees (Egbunike and Imade 2017). They often operate at micro level, trading amongst themselves and their clientele, with generally low volumes of trade (Ruiter et al. 2017). Given increasing global connectivity and the proliferation of information technologies, these traders have embraced electronic commerce to enhance their economic activities (Croke et al. 2020). Thus, like any other sector, they are vulnerable to cybersecurity threats (Chigada and Madzinga 2021). While empirical studies have been conducted on the cybersecurity vulnerabilities faced by big firms, there are limited empirical studies focusing on small-scale African migrant traders and the cybersecurity vulnerabilities they face. This study aimed to fill this gap.

## Related literature review

The COVID-19 pandemic has transformed the world, with information communication technology reshaping economies, nations, businesses, and commerce. This has resulted in cyber threats becoming an ever-present reality (Syed 2020). Existing defensive mechanisms have proved inadequate to address them and incidents of hacking have dominated the news media (Gourisetti et al. 2020). The methods employed include ransomware, malware, spam emails, and malicious domains (Khan et al. 2020). Moreover, cybercriminals are using COVID-19 disinformation as another weapon to destabilise businesses and financial institutions locally and globally (Crisanto and Prenio 2020). According to Interpol (2020), cybercriminals use fake news and disinformation as a lure to attack information systems, computers networks, data servers and mobile devices.

As a result, cybersecurity research began to gain traction as academics and practitioners sought to develop approaches and tools that are able to detect and prevent cyber threats early and before they destroy systems (Chigada and Hirschfelder 2017). Cybercriminals have taken advantage of the shift in global attention to health and other strategies to curb the spread of COVID-19 (Carías et al. 2020). Irrespective of their size, businesses that operated online through less reliable and unsecured Internet connections have encountered cyber risks (Rao et al. 2021).

As global trade becomes increasingly interconnected, the chances of exposure to cybercrime increase in the same proportion (Ruvin et al. 2020). However, it is important to note that most cyberattacks and threats are perpetrated by human beings. This implies that human behaviours and ethical conduct are significant determinants of vulnerabilities that make entrepreneurs susceptible to cyberattacks (Pandey et al. 2020). Hence it is essential to discuss the vulnerabilities of small-scale traders while working remotely because with the increasing global connectivity and the proliferation of information technologies, small-scale traders have embraced remote working style to enhance their economic activities (Croke et al. 2020).

According to Bentley et al. (2016), socio-technical systems play a vital role in the proliferation of flexible work such as remote working. With socio-technical systems, people are allowed to work with technology in a flexible way that benefits them and advances organisational goals (Ofusori and Subramaniam 2021). Reuschke et al. (2021) contended that remote working provides the flexible working style for most traders which has changed their socio-economic life as it enables them and other entrepreneurial business to transact online across different locations and time zones. However, there are technical security issues such as hacking, phishing and data interception, to mention a few, that come with the social-technical systems. These threats have the potential to expose businesses to cyberattacks owing to their association with people while working remotely. According to Matli (2020), the COVID-19 pandemic accelerated remote working due to government restrictions and other regulations that limited in-person engagement. As a result, entrepreneurs were compelled to work and transact online with limited security measures as this was a new phenomenon that the entire world was adjusting to.

According to Khan et al. (2020), online transacting exposed traders to cybercriminal activities because most of them had not kept up with the latest cybersecurity technologies that would protect their systems and networks. This implies that with the high cost of data and limited secured Wi-Fi networks, entrepreneurs and their employees had to access unsecured Wi-Fi networks to do their transactions, thereby being susceptible to hackers and other related cybersecurity threats such as phishing (Joel 2021), malware attacks (Tu et al. 2019), ransomware and spam emails (Chigada and Madzinga 2021). With the understanding of vulnerabilities of small-scale traders while working remotely, it is also appropriate to establish the contextual aspects for small-scale African migrant traders in Southern African.

Africa is often conceived of as a continent characterised by mass migration and displacement caused by poverty, violent conflict, and underdevelopment, as well as environmental stress and food insecurity (Flahaux and De Haas 2016). This scenario has been exacerbated by the COVID-19 pandemic. Intra-African trade increased as countries eased their COVID-19 regulations and travel restrictions in a bid to stimulate local and regional economies (Dovi 2018). Moreover, the prospects of economic recovery, coupled with social transformation and improved immigration policies, have promoted increased intra-African migration, especially in relation to trading, as most businesses were decimated by the pandemic (Morsy et al. 2021). African traders migrating to Southern Africa hail from West Africa (Nigeria, Cameroun, Gambia, Ghana, Senegal), East Africa (Uganda, Kenya, Tanzania, Ethiopia, Somalia) and from within Southern Africa (Lesotho, Mozambique, Angola, Zimbabwe, Zambia, eSwathini, Botswana and Namibia) (Nkrumah 2021).

The economic and industrial development in certain sectors, such as the mining, manufacturing, and ICT sectors in South Africa, has attracted skilled and unskilled African migrants from around the continent (Plagerson et al. 2019). According to the United Nations Statistics Division (UNSD), an estimated 2.9 million migrants resided in South Africa in mid-year 2020. Other “pull” factors include better management of the COVID-19 pandemic, relatively functional health infrastructure, rapid economic recovery, and higher overall socio-economic development (Oladele and Vieyra-Mifsud 2021). However, the “push” factors that deter intra-African migration include hostility towards migrants, with sporadic xenophobic incidents in South Africa, political persecution, high levels of unemployment, limited access to basic social services (housing, health, and education) and the challenge of accessing credit for business (Pasara 2020). However, these factors have not halted the wave of Africans migrating to the region to set up businesses (Croke et al. 2020).

Intra-continental migration will continue as countries in the region recover steadily from the effects of the pandemic (Bello and Matthew 2021). Most African traders engage in small-scale trading, often retail, within and across borders (Bouët et al. 2021). However, whilst the region has viable economic prospects that promote meaningful entrepreneurial activities, the COVID-19 pandemic and related travel restrictions have meant that entrepreneurs are forced to transact digitally (Mataba and Ismail 2021). This transition intensified as waves of the pandemic emerged and prompted governments to restrict trading activities to curb transmission (Ndzendze and Monyae 2019). Increased use of digital platforms for transactions has further exposed traders to cyber threats and risks (Banga et al. 2021). Most small-scale African migrant traders do not protect their information systems from such risks, resulting in the loss of data privacy and income (Reva 2020). This calls for a multi-pronged approach involving the traders themselves, Internet service providers (ISPs), locally based migrant business associations, governments, and business chambers to create multiple support mechanisms to assist traders to cope with the changing economic and trading environment in the diaspora. The discussion on related literature is incomplete without explaining the effects of cyber threats and attacks on small-scale traders. Hence, it is essential to establish the effects of cyber threats and attacks on small-scale traders.

Small-scale traders are susceptible to cybersecurity threats and attacks as these are transmittable via mobile devices which they often use for their transactions. While there are no official data on the financial losses incurred by small-scale traders, the World Bank (2019), World Health Organization (WHO) (2020), World Economic Forum (2019), Centers for Disease Control (CDC) (2020) and Cybersecurity Ventures (2020) predicted that cybercrime incidents would cost the world US$6 trillion by 2021, up from US$3 trillion in 2015 (Chigada and Madzinga 2021, p. 8). This highlights how costly such vulnerabilities are, as well as the need for an effective response. Whilst the financial losses that small-scale traders incur remain largely undocumented, they have become easy targets due to their size and lack of financial resources to hire skilled personnel to protect their information technology (IT) systems from data breaches and leakage leading, among other things, to the loss of client information. Data leakage refers to the transfer of sensitive or classified information from a secured system to an unauthorised third party (Bertrand et al. 2020).

## Research model

A research model is an established scientific framework that explains various variables (constructs) surrounding a given phenomenon and their interrelationship (Lewis and Thornhill (2011). This study reviewed related research models with the aim of conceptualising an integrated framework. Technology threat avoidance theory (TTAT), protection motivation theory (PMT) and security perception theory are the three models that contributed to the conceptualisation of the framework used in this study.

**Fig. 1:**
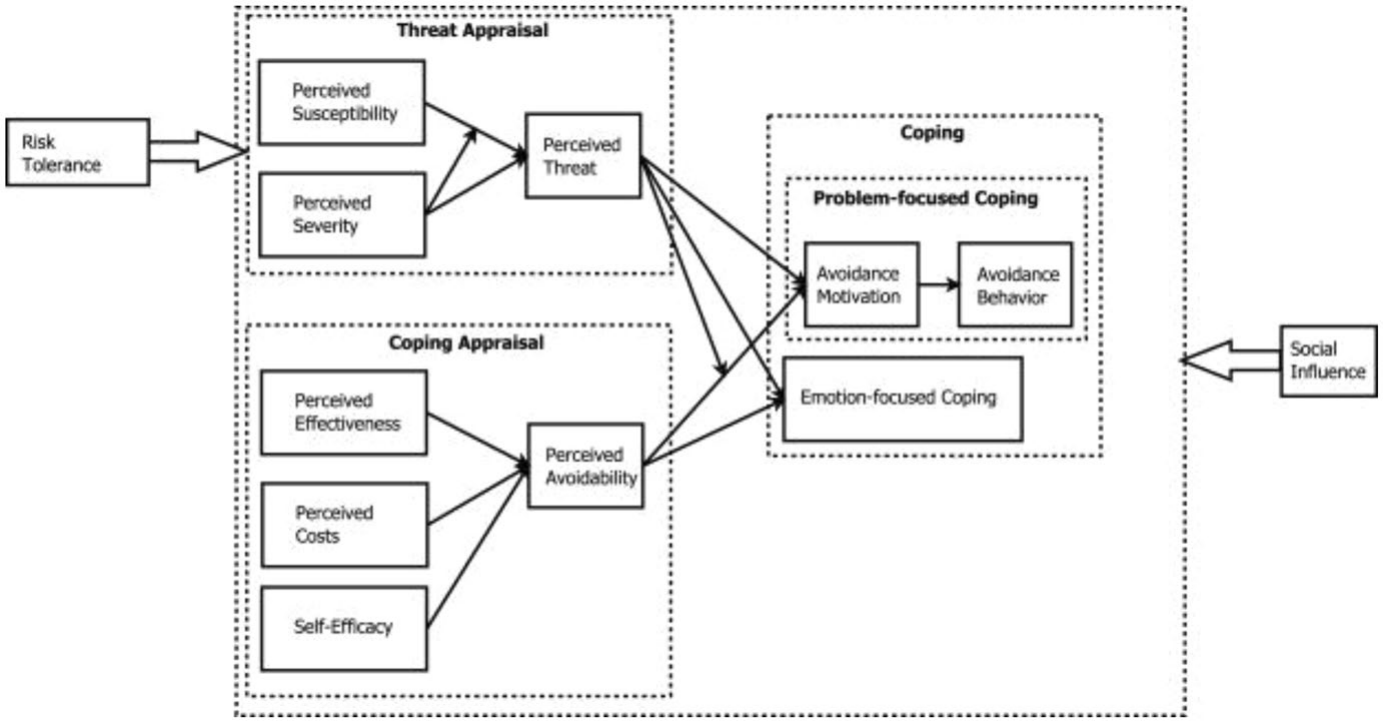
Technology threat avoidance theory (Liang and Xue 2009)

The first theory is ***technology threat avoidance theory (TTAT)***. TTAT was proposed by Liang and Xue (2009) to explain the behaviour of individual IT users that engage in threat avoidance behaviours (Fig. 1). TTAT has been found to help explain user avoidance behaviour through cybernetic and coping theories (Liang and Xue 2009). Drawing from cybernetic theory and coping theory, TTAT delineates the avoidance behaviour as a dynamic positive feedback loop in which users go through two cognitive processes, threat appraisal and coping appraisal, to decide how to cope with IT threats (Carver and Scheier 1982; Edwards 1992). Threat appraisal proposes that users' threat perception is determined by two factors: the perceived susceptibility (users’ assessment of the level of threat’s occurrence) and the perceived severity (threat's negative consequences). Liang and Xue (2009) modelled threat susceptibility and threat severity as having both direct and an interactive effect on perceived threat. Likewise, Carpenter et al. (2019) revealed that the relationships between susceptibility, severity, and threat perceptions is highly significant. In the context of this study, these factors proved helpful in measuring how vulnerable the small-scale African migrant traders are to security risks while using digital technologies in transacting business (Fig. 4).

With coping appraisal, the users consider three factors to evaluate how avoidable the threat can be made by a safeguarding measure: the costs of the measure, the effectiveness of the measure, and users' self-efficacy of taking the measure. However, Siponen et al. (2014) affirm that self-efficacy is the most vital predictor of intention to comply with a behaviour. According to Liang and Xue (2009), self-efficacy has a positive and significant impact on individuals’ intention to comply with the policies and procedures. In the context of this study, self-efficacy was helpful in measuring the individuals’ (i.e. small-scale African migrant traders) belief in executing security measures to adhere to information security policies and procedures (Fig. 4). TTAT posits that users decide how to cope with IT threats by engaging in problem-focused coping or emotion-focused coping (Lazarus and Folkman 1984; Lazarus 1966). In problem-focused coping, users are motivated to avoid malicious IT when they perceive a threat and believe that the threat is avoidable by taking safeguarding measures. However, if users believe that the threat cannot be fully avoided by taking safeguarding measures, they would engage in emotion-focused coping.

It is should be noted that the constructs of perceived susceptibility and perceived severity were used to measure perceived threats, illustrated in Fig. 1. Likewise, this study adopted these constructs to measure cybersecurity threat, as shown in Fig. 4. Also, while the construct of self-efficacy was used to measure perceived avoidability, as in Fig. 1, it was adopted to measure intention to comply to security threats, as in Fig. 4. However, the other TTAT constructs are not relevant to this study and could not be adopted.

The second theory is the ***protection motivation theory (PMT)***. PMT aims to understand the effects of fear appeals and how people cope with them (Rogers 1975). PMT has been widely used in health sciences and only a handful of researchers have tested the theory in the field of technology. Ofusori (2019) used PMT to investigate the link between individual and organisational practices to mitigate security threats arising from BYOD-enabled banking institutions in Nigeria. Siponen et al. (2014) integrated PMT with the theory of reasoned action (TRA) and cognitive evaluation theory to explain employees’ adherence to information security policies. Putri and Hovav (2014) further combined PMT with reactance theory and organizational justice in explaining employees’ compliance with BYOD security policy.

**Fig. 2:**
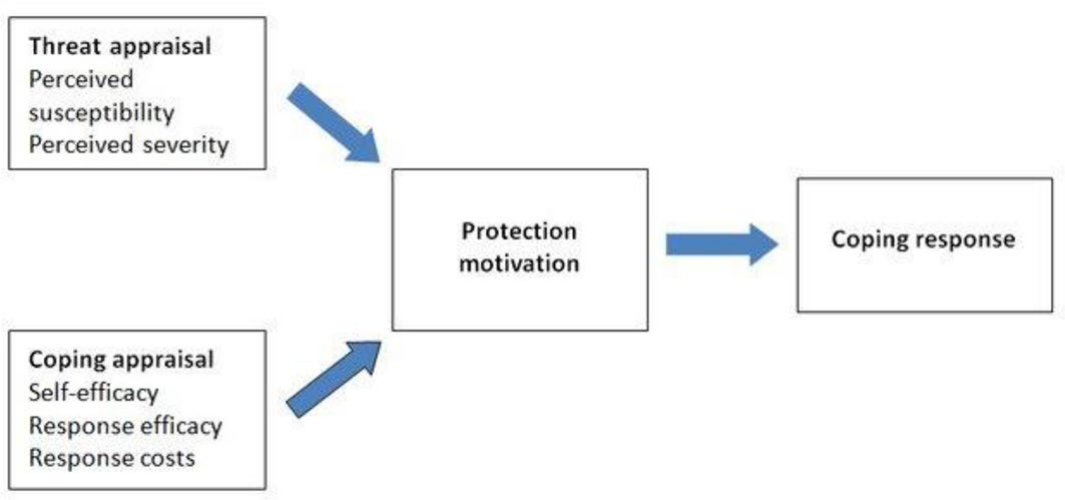
Protection motivation theory (Rogers 1975)

PMT states that the motivation to take protective action is as a result of threat appraisal and coping appraisal (Fig. 2) (Maddux and Rogers 1983). In threat appraisal, users assess the level of risk that results from falling victim to a security threat (perceived susceptibility) and how serious it is (perceived severity). According to Maddux and Rogers (1983), PMT suggests that individuals assign a level of severity to the threat (perceived severity), while simultaneously gauging the likelihood that the threat will affect them personally if no measures are taken to counter the problem (perceived susceptibility). In the context of this study, these are measures of how vulnerable the small-scale African migrant trader is to security risks while using digital technologies in transacting business.

The coping appraisal is determined by three factors, namely self-efficacy (to what extent the individual has confidence in their ability to carry out the action), response efficacy (to what extent the individual thinks the action will deal with the threat), and response costs (the perceived disadvantages of undertaking the action). Self-efficacy is the most powerful predictor of intention to comply with a behaviour (Siponen et al. 2014). However, Maddux and Rogers (1983) affirmed that self-efficacy is as an integral construct for forming protection motivations. They proposed that individuals must believe the response mechanism will effectively protect them from a threat, but must also believe in their ability to implement the mechanism (Boysen 2018). In the context of this study, self-efficacy refers to small-scale African migrants’ belief that they can apply and adhere to information security policies and procedures (Fig. 4).

It is important to note that while the constructs of perceived susceptibility, perceived severity and self-efficacy were used to measure protection motivation, as in Fig. 2, this study adopted these constructs (perceived susceptibility and perceived severity) to measure cybersecurity threat and the construct of self-efficacy to measure intention to comply to security threats (Fig. 4). This is because these constructs (perceived susceptibility, perceived severity and self-efficacy) are suitable in explaining the individual perceptions of security risks and the likelihood of experiencing them in relation to using digital technologies in transacting business (Fig. 4). The other PMT constructs are not relevant to this study; hence they were excluded.

**Fig. 3:**
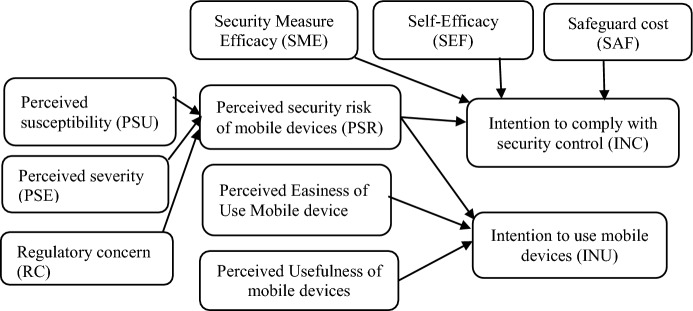
A security risk perception model for the adoption of mobile devices (Alexandrou and Chen 2019)

The third theory is the ***security risk perception model*** proposed by Alexandrou and Chen (2019). This theory focuses on how individuals perceive the risks associated with mobile devices usage. It posits that people have specific security beliefs that could indirectly impact their behavioural intentions to use mobile devices. This compels them to have security controls when using mobile devices (Alexandrou and Chen 2019). The security perception model is determined by three major factors: perceived security risk of mobile devices, intention to comply with security control and intention to use mobile devices (Fig. 3) (Alexandrou and Chen 2019; Vance et al. 2014). According to Alexandrou and Chen (2019), the “perceived security risk of mobile devices” is evaluated by estimating the chances of the mobile device being compromised by security threats (perceived susceptibility), the seriousness of the compromise (perceived severity), and the regulatory concerns (guidelines and specifications relevant to the mobile device). Likewise, “intention to comply with security control” is measured by how the security threat is examined (security measure efficacy), how the individual executes security policies (self-efficacy), the costs incurred in protecting the mobile device from security threats (safeguard cost), and users’ perceptions of the likelihood of their mobile devices being compromised (perceived security risk of mobile devices). Also, “intention to use mobile device” is determined by three factors, namely perceived ease of use of mobile device, perceived usefulness of mobile device and perceived security risk of mobile devices. Drawing upon the constructs of the security perception model highlighted above, this study adopted the constructs that are relevant to the study. This included perceived susceptibility, perceived severity, perceived security risk of mobile device, security measure efficacy, self-efficacy and intention to comply with security controls. This is because these constructs were suitable for the study as they enabled the researcher to investigate how cybersecurity threats and vulnerabilities impact small-scale African migrant traders in Southern Africa. Other constructs were excluded because they are not relevant to the study.

Based on the discussion on the three theories provided above, the conceptual model for this study was derived.

This study adopted constructs from TTAT, PMT and security perception models to develop an integrated framework called Perceived Security Compliance Framework (PSCF). These constructs were adopted due to their ability to explain individual perceptions of security risks and the likelihood of experiencing them in relation to using digital technologies in transacting business. Moreover, the models have been widely accepted and used to predict security threats due to their strong predictive power (Vance et al. 2014).

It is important to note that Liang and Xue (2009) in their study, while using TTAT, the focus of the problem was to measure the behaviour of IT users that engage in threat avoidance. Likewise, Dang-Pham and Pittayachawan (2015), when using PMT, the focus of the problem was to investigate students’ intention to avoid malware in a BYOD-enabled Australian University. Also, Alexandrou and Chen (2019), while using the security risk perception model, the focus of the problem was to understand how medical practitioners perceive the risks associated with mobile devices especially with regard to BYOD. However, the focus of this study differed from the three referred to above here, and was to investigate individual perceptions of security risks and the likelihood of experiencing them in relation to using digital technologies in business transactions.

**Fig. 4:**
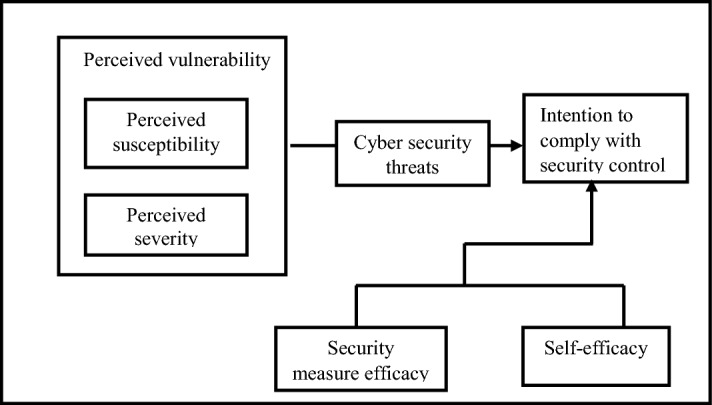
Perceived security compliance framework (Authors’ own)

The PSCF (Fig. 4) argues that a person's intention to adopt security controls is determined by their belief that they are at risk (perceived susceptibility), the seriousness of the risk (perceived severity), the risk (cybersecurity threat), how the security threat is examined (security measure efficacy), and how the individual executes security policies (self-efficacy). While perceived susceptibility refers to how an individual evaluates the chances of falling victim to a security threat, perceived severity refers to how they evaluate/estimate the seriousness of a security threat. The model predicts that people who are highly susceptible to cyberattacks may fall prey to cybersecurity threats. Hence, the individual (small-scale African migrant trader) needs to engage in security behaviour and comply with security controls that reduce the risk of exposure. Such behaviour includes security measure efficacy and self-efficacy. Security measure efficacy focuses on how an individual handles security threats and how they cope or react to them in the workplace. It also examines how comfortable people are in executing the security policies required when using mobile devices in the workplace. Lastly, self-efficacy investigates how the small-scale African migrant traders execute security policies.

## Research methodology

This qualitative study adopted a non-probability sampling technique as the researchers targeted a group of small-scale African migrant traders who traded across Southern Africa and were regular users of digital platforms for their entrepreneurial activities. The researchers were not seeking to generalise the findings to the overall population of small-scale African migrant traders, but to document their exposure to and experiences of cybersecurity threats (Saunders et al. 2018). Non-probability sampling also saves time and is cost-effective. Purposive sampling was adopted as the researchers sought to obtain in-depth information on small-scale African migrant traders and their enterprises’ exposure to and experience of cyber threats due to their use of digital platforms whilst trading. This non-probability technique was deemed appropriate as it yielded a sample that was believed to be representative and convenient.

Snowball sampling was used to identify respondents. The first respondent was initially known by researchers through their own personal connections as African migrants living in South Africa and through this association they were able to identify and be referred other respondents for the study. Furthermore, these respondents referred other respondents until the sample size was achieved.

In this technique, a small number of individuals are identified to represent a population with specific common characteristics. This technique was appropriate as it enabled the researchers to identify small-scale African migrant traders based on their shared experiences. The sample size was 21 made up of 15 African migrant small-scale traders and six key informants who were representatives of organised African migrant business associations (refer Tables 1 and 2). Based on the conceptual model, an interview protocol was designed with five main parts (refer Table 3).

**Table 1:** Small-scale African migrant traders interviewed

Trading countries	Nature of business	No. of years as a small-scale trader	Participant code
Nigeria	Import and export of agricultural products, cell phone accessories and Nigerian fabric	15	Participant 1
Ghana	Import and export of agricultural products and cell phone accessories	15	Participant 2
Guinea	Guinean fabric	8	Participant 3
Senegal	Import and export of agricultural products, cell phone accessories and Senegalese fabric	15	Participant 4
Liberia	Agricultural products, cell phone accessories and Liberian fabric	15	Participant 5
Cameroon	Import and export of Cameroonian fabric and agricultural products	15	Participant 6
Uganda	Import and export of Ugandan fabric	5	Participant 7
Kenya	Import and export of curios, Kenyan fabric and agricultural products	10	Participant 8
Tanzania	Import and export of curios, Tanzanian fabric and agricultural products	5	Participant 9
Ethiopia	Import and export of cell phone accessories and agricultural products	10	Participant 10
Zimbabwe	Import and export of curios and Zimbabwean fabric	10	Participant 11
Somalia	Import and export of cell phone accessories and textiles (blankets)	12	Participant 12
Eritrea	Import and export of cell phone accessories and agricultural products	15	Participant 13
Lesotho	Import and export of agricultural products and traditional ceramic products	10	Participant 14
Mozambique	Import and export of clothing (sportswear and shoes) and cell phone accessories	14	Participant 15

**Table 2:** Key informants representing African migrant business associations

Business association	Participant code
Somalian business network	Participant 16
Nigerian business network	Participant 17
Kenyan business network	Participant 18
Lesotho business network	Participant 19
Cameroon business network	Participant 20
Mozambique business network	Participant 21

**Table 3:** Interview protocol

Items	Details	Interviewees
Demographic characteristics	Name of country Nature of business No. of years as a trader	Small-scale African migrant traders
Perceived vulnerability	Questions related to perceived susceptibility to cybersecurity threats Questions related to perceived severity of cybersecurity threats	
Security measures efficacy	Questions related to cybersecurity measures	
Self-efficacy	Questions related to awareness of cybersecurity threats	
Intention to comply with security protocols	Questions related to support mechanisms	Representatives of African migrant business associations

Primary data was collected by means of semi-structured and key informant interviews. All the interviewers were conducted in English Language as all respondents were conversant with it. The interviews explored specific topics and solicited the traders’ individual views on and experiences of transacting via digital platforms and their businesses’ degree of exposure to cyber threats. These data collection tools provided critical in-depth information that could be conformed via triangulation. All the interviews were open-ended in nature, which enabled a free-flow conversation and allowed the researchers to further probe issues that emerged.

Secondary data was collected through search engines such as PubMed, Google Scholar and Scopus to identify relevant articles, including research reports and websites to better understand the cybersecurity threats and vulnerabilities associated with small-scale African migrant traders in Southern Africa. Table 4 presents the mapping of relevant extracted articles with the number of articles from the various sources. It shows the number of journals, research reports, books and websites that cover the phenomenon of cybersecurity threats and vulnerabilities among small-scale traders in Southern Africa.

**Table 4:** Relevant sources of extracted articles

Source	Repository	Number of articles	Indexed
Journals	Transnational Corporation Review	1	ESCI, Scopus, ABDC, EBSCO
	Comparative Migration Studies	2	Scopus, DOAJ, Google Scholar
	World Development	1	Web of science, Clarivate Analytics
	Technium So. Sci. J	1	DOAJ, EBSCO, Google Scholar
	Africa Review	1	Scopus, OCLC
	African Journal of Economic Review	1	Google Scholar
	Security Journal	1	Scopus, ProQuest, EBSCO
	Computers in Human Behaviour	2	Scopus, PsycInfo, PsycLit
	Frontiers in Big Data	1	Scopus, Web of Science, Clarivate
	European Societies	1	Scopus, ISI and OCLC
	IEEE Access	1	Scopus, EBSCO, Google scholar
	South African Journal of Information Management	1	EBSCO, ProQuest, DOAJ
	Enterprise Information Systems	1	Scopus, Web of Science
	Journal of Criminology	1	Scopus, PsycInfo
	African Journal of Criminology & Victimology	1	Scopus, PsycInfo
	International Journal of Accounting Research	1	EBSCO, Publons
	International J. of Information Security Science	1	EBSCO, DOAJ, WorldCat
	Future Generation Computer Systems	2	Scopus, Web of Science, INSPEC
	Journal of Financial Economics		COREJ, Social Sciences Citation Index
	Computer and Security	1	INSPEC, Scopus
	Journal of Experimental Social Psychology	1	Scopus, PsycInfo, PsycLit
	Computer Fraud and Security	1	Scopus, INSPEC
	International Journal of Advanced Research in Computer Science	1	EBSCO, ProQuest, Google Scholar
	Journal of Global Operations and Strategic Sourcing	1	ProQuest, INSPEC, Scopus
	Entrepreneurship and Sustainability Issues	1	DOAJ, Web of Science, Clarivate
	The Journal of Psychology	1	ProQuest, Scopus, PubMed, Medline
	Journal of Security and Sustainability Issues	1	Scopus
	Information and Management	1	INSPEC, Social Science Citation
	Journal of the Midwest Association for Information Systems	1	Social Science Citation Index expanded,
	Journal of the Association for Information Systems	1	Social Science Citation Index expanded
Books	Data Analytics and Decision Support for Cyber Security	1	Springer
	Ethical Hacking Techniques and Countermeasures for Cybercrime Prevention	1	IGI
	Online Social Networks Handbook of Research on Cyber Crime and Information Privacy	1	IGI
Research reports	Current Psychiatry Reports	1	ProQuest, Scopus, EBSCO, Medline
	ISS Africa Report	1	Not indexed
	Internal Food Policy Research Institute	1	
	World Bank Policy Research Working Paper	1	
	Sauti Africa report	1	
	African Renewal	1	
Websites	www.bizcommunity.com	1	
	www.cdc.gov	1	
	www.trendmicro.com	1	
	www.swivelsecure.com	1	
	www.interpol.int	1	
	www.weforum.org	1	
	www.who.int	1	
	www.wider.unu.edu/	1	

Once the interviews had been conducted, the transcription process commenced. Each interview tape was converted into a readable transcript. This time-consuming process was undertaken cautiously to ensure that no detail was left uncaptured. Thereafter, substantive parts of the transcript that related to the research questions, as well as new topics or issues, were classified and coded in themes. The coding process involved the identification of passages in the texts that related to each other and linked to the research questions and other data collected for the study.

Axial coding following the open coding, where the “…relationships between the categories of data that have emerged from open coding” (Saunders et al. 2018) were identified. At this stage, the coded data was reduced and re-classified into broader categories, which are more conceptual than literal. Stated differently, the initial codes were re-organised into broader concepts. Summative content analysis followed axial coding. This involved analysing manifest content, as well as latent meanings and themes (Zhang and Wildemuth 2009). In this phase, the researchers searched for connectivity of themes, and relationships between and within sub-categories. Thereafter, the coded data was further processed into themes in preparation for detailed thematic analysis. The process of open coding yielded 12 themes (5 major themes, and 7 sub-themes as shown in Tables 5 and 6). Additionally, the responses used for the findings were uniquely identified using codes for each of the major theme categories. For example, PSU refers to Perceived susceptibility.

**Table 5:** Themes and sub-themes that emerged from interviews with small-scale African migrant traders

Variables	Major themes	Sub-themes
Perceived vulnerability	Perceived susceptibility (PSU)	Unprotected networks
	Perceived severity (PSE)	Uncontrolled access
Security measure efficacy	Cyber-protection (CP)	Negligence
		Inadequate security measures
Self-efficacy	Awareness (AW)	Lack of awareness

**Table 6:** Major theme and sub-themes that emerged from key informant interviews

Variable	Major theme	Sub-themes
Intention to comply with security control	Support mechanisms (SM)	Support mechanisms from ISPs
		Support mechanisms from business associations

To ensure reliability, the researchers piloted the research instruments with two independent researchers to ensure that the questions matched the answers. Any contradictions were addressed to ensure that appropriate and trustworthy responses would be solicited. In terms of validity, the researchers ensured that the research instruments, the data collection process and the study’s findings accurately reflected the data collected and corresponded with the theoretical concepts in the research topic.

Furthermore, ethical considerations were taken into account where respondents consented to their voluntary participation in the study. The researchers approached each small-scale African migrant trader individually at different locations and different times. These individuals were identified to represent a population with certain specific common characteristics. The aims and objectives of the study were explained as well as the identity of the researchers and how the respondents had been selected. All the information provided by the participants was treated as confidential and was not divulged to any third party. Pseudonyms are used in this paper to conceal the participants’ identities. All digital recordings including field notes were safely stored throughout the fieldwork period.

## Data analysis and findings

The section presents results of data analysis emerging out of two interviews. The first interview aimed to identify common cybersecurity vulnerabilities and threats experienced by small-scale African migrant traders in Southern Africa. The second interview aimed to evaluate the support mechanisms available to small-scale African migrant traders by the business associations.

The first interview is used to collect data related to three variables, namely; perceived vulnerability, security measure efficacy and self-efficacy. For perceived vulnerability, two major themes were identified namely; perceived susceptibility and perceived severity, while unprotected networks and uncontrolled access emerged as sub-themes, respectively. Similarly, with security measure efficacy, cyber protection emerged as the major theme, while negligence and inadequate security measures emerged as the sub-themes. Likewise, with self-efficacy, awareness emerged as the major theme, while lack of awareness emerged as the sub-theme.

The second interview is used to collect data related to intention to comply with security control. With “intention to comply with security control”, support mechanism emerged as the major theme, while support mechanism from ISPs and support mechanism from business association emerged as the sub-themes.

Given below is the findings emanating from the analysis carried out using the data collected from the first interview. The responses below were selected because they represent those of other respondents and are well presented. The themes that emerged from the analysis of the data from the first interviews were subsequently classified into sub-themes, as shown in Table 5.

### Perceived vulnerability

Perceived vulnerability is an assessment to measure the scale of a threat's exposure done by an individual (Albladi and Weir 2020). According to Alexandrou and Chen (2019), individuals’ intention to comply with security control get affected by their beliefs of being at risk (perceived susceptibility) and the seriousness of the risk (perceived severity). Hence, an effort was made to measure susceptibility and severity through a key question for each category. The key question was guided by several related questions for better response.

#### Major theme: perceived susceptibility (PSU)

The key question for this section was: To what extent are small-scale African migrant traders susceptible to cyber-related attacks? The study aimed to establish the extent of cyberattacks associated with small-scale traders and the platforms used as well as access control mechanisms available on those platforms. The major themes from the interviews are presented in this section.

##### Sub-theme: unprotected networks

Mobile technology has enabled businesses to connect across information networks and digital platforms. This has exposed business entities to threats and risks that were previously unknown (Iyaloo 2019). A trader from a Southern African country observed:

I’ve seen … cyberattack activity increasing, especially through the mobile devices… Most of us [entrepreneurs] have not considered protecting our systems from such attacks …making us susceptible to the attacks which severely affects our businesses and their potential to grow in the region…. (Participant 7).

Another stated:

I was hacked while transacting online…purchasing my stock from a supplier abroad…I wasn’t aware that I was exposed to cyber criminals. (Participant 10).

And a third stated:

I normally use my business partner’s device to transact with clients…until I discovered that my account has movements that I couldn’t explain, and I realised that I’m under cyberattack of some sort.… (Participant 2).

In line with the above statements, it is apparent that the small-scale traders’ mobile devices are vulnerable to cyberattacks (refer PSU, Participants 2, 7, and 10). This is because they are connected to secure and unsecure networks where the security policies differ (Ofusori and Subramaniam 2022). This interconnectivity increases the likelihood for unauthorised access to confidential and private information, scam, misuse or abuse (Zinkus et al. 2021). According to Iyaloo (2019), mobile devices that are connected to wireless networks are more vulnerable to cyberattacks because the wireless connections are difficult to secure and hence, hackers can intercept and control the traffic. Furthermore, every connection has its own potential security susceptibility and allows for easy eavesdropping (Iyaloo 2019). Joel (2021) affirmed that in 2018, 71% of ransomware attacks targeted small-scale businesses, with an average ransom demand of $116,000. In this study, the vulnerability of traders’ mobile devices to cyberattack may represent perceived susceptibility (an assessment that measures the scale of a threat's exposure done by an individual).

#### Major theme: perceived severity (PSE)

The key question for this section was: To what extent does access to digital trading platforms by third parties expose traders to cybersecurity threats? The major themes from the interviews are presented in this section.

##### Sub-theme: uncontrolled access

All the traders that participated in the study had no control measures to guide their interaction with other traders through their business networks. These networks are thus exposed to cybercriminals. The following are some of the responses:

I’ve never thought about having controlled access to my business networks by my suppliers and other stakeholders that I interact with daily.… (Participant 1).

I work with two computers systems belonging to me and my business partners and we transact with the systems daily [not knowing] that they are exposed to cyber criminals that monitor our online transactions. … (Participant 5).

Our business is not covered from cyber hacking and I didn’t know how dangerous it was to have a business unprotected from cyber criminals through software that protects my data …and transactions. (Participant 9).

The above statements reveal that the traders do not have control measures that can guide their interaction with other traders through their business networks (refer PSE, Participants 1,5 and 9). The implication of this is that, not only are they vulnerable to cyberattacks but the severity of the attacks will be critical due to lack of security measures. Supporting this finding, Burk and Prince (2019) argued that the Internet is full of hackers and malicious software that try to access any protected and unprotected systems. Hacking compromises digital devices and networks through unauthorised access to an account or computer system (Yadav and Mestry 2021). According to Ofusori (2019), mobile devices are more susceptible to cyberattacks if appropriate security measures are not put in place. In the context of this study, the negative consequences of the vulnerability of traders’ mobile devices to cyberattack represent perceived severity.

### Security measure efficacy

Security measures efficacy refers to the traders’ ability to protect their information systems from cybersecurity threats (Joel 2021). An effort was made to measure cyber protection through a key question. The key question was guided by several related questions for better response.

#### Major theme: cyber protection (CP)

The key question for this section was: To what extent do small-scale African migrant traders protect their information systems from cyber-related attacks? The study aimed to establish the small-scale traders’ level of cyber protection. None of the traders interviewed had adequate security measures in place as part of their overall business strategy to protect themselves from cyberattacks, making them vulnerable to such attacks. The major themes from the interviews are presented in this section.

##### Sub-theme: negligence

The business owners acknowledged that they have been negligent and complacent in underestimating the impact of cybercrime on their business operations, especially in terms of financial losses. However, none could quantify how much they had lost financially due to cybercrime. A trader observed:

It is incomprehensible that we [business owners] sometimes get attacked…and our accounts infiltrated by cyber criminals…resulting in loss of revenue and data (business data). … (Participant 6).

Another trader responded:

We do not know how much we lose financially as result of cyberattacks…somehow we don’t pay attention to this.… (Participant 4).

Similarly, a respondent remarked:

You see most of us [business owners] just enjoy the interconnectedness but we’re ignorant of the risks we are exposing our enterprises to…. (Participant 11).

The findings above suggest the traders are nonchalant (refer CP, Participant 4) and ignorant (refer CP, Participant 11) of the impact of cybercrime on their business operations. This ignorance about the impact of cybercrime has led to financial losses of among others (refer CP Participant 6). Several studies have shown the negative effect of cybercrime on business operations in terms costs (mainly financial losses) (Klahr 2017; Johns 2020; Furnell et al. 2020; Ajayi 2016). Hence, it is essential that the traders are able to protect their information systems from cybersecurity threats (Joel 2021). In the context of this study, the cost incurred in protecting the mobile device from security threats represent security measure efficacy.

#### Sub-theme: inadequate security measures

None of the traders had adequate security measures to limit their exposure to cyber-security threats. Their business enterprises were thus vulnerable to hackers, who remain in their networks for long periods of time without detection. The following are some of the responses from the participants:

We are unaware that hackers remain on our networks and continue their illegal activities…we do not know we need to enable the firewalls on our computer systems.… (Participant 3).

I didn’t know that small businesses like mine could be attacked by cyber criminals because our systems (trading)…are exposed because we don’t have software to protect our machines (laptops) and trading accounts. (Participant 8).

Trading with my machines (laptops) has exposed my business to all kinds of risks…I never knew I can enable the firewall on my system as part of my security measures.… (Participant 13).

All the above comments made by the respondents suggest that the traders do not enable the security software on their systems (refer CP, Participants 3 and 13). Supporting this finding, Bitton et al. (2018) affirmed that while a lack of proper security measures for mobile devices is a vulnerability, the risk is magnified when people are unaware of the importance of the security measures. Shazmeen and Prasad (2012) asserted that some security measures must be adopted and enabled to reduce the possibility of a security incident. In the context of this study, the cost incurred in procuring the security software represents security measure efficacy.

### Self-efficacy

Self-efficacy refers to an individual’s perceptions that they can acquire and utilise cognitive and other resources to improve their knowledge and understanding of cybersecurity threats and countermeasures (Bandura 1977). This study made an effort to measure security risks awareness through a key question. The key question was guided by several related questions for better response.

#### Major theme: awareness (AW)

The key question for this section was: To what extent are small-scale African migrant traders aware of the security risks associated with the use of digital platforms for trading? The respondents demonstrated limited awareness of the security risks associated with digital interconnectedness as well as mitigation strategies that they need to safeguard their businesses from cyberattacks.

##### Sub-theme: lack of awareness

As a result of lack of awareness, none of the traders had a mitigation strategy to prevent illegal access to the platform that they use for transactions. They also seemed to be unaware of why such knowledge is important for their business. The following are some of the responses:

I never thought it is important for me to be aware of the risks because I took it for granted. (Participant 9).

I always heard about it but I never took it seriously. (Participant 15).

Even though I knew about the risks that my business was exposed to, I never had a plan that will protect my online transactions. (Participant 12).

I have not always bothered to [learn about] risks associated with digital platforms, [and the] pros and cons of online transactions because I never thought it was necessary for a small trader like me. (Participant 14).

I never thought that a small business like mine would require any sophistication like having a strategy and plan. I only deal with … making money. (Participant 1).

The findings above imply that there is a lack of adequate awareness and comprehension amongst the traders on the severity and vulnerability of using mobile devices in a work context (refer AW, Participants 1, 9, 12, 14 and 15). Corroborating these findings, Fuentes (2020) revealed that small businesses often have less awareness of threats and less time and resources to protect their devices from cybersecurity threats. Supporting this claim, Ofusori (2019) maintained that security awareness on mobile devices is so poor that it leaves businesses vulnerable to security threats. Unless traders take the measure into consideration, it makes them easier targets by hackers as small traders in comparison to bigger and more established business entities which have the necessary security measure in place. Hence, it is important the traders are enlightened on the cyber threats and the effects. In the context of this study, the ability of the traders to acquire personal knowledge such as attending a cybersecurity awareness workshop represents self-efficacy.

Given below is the findings emanating from the analysis carried out using the data collected from the second interview. The responses below were chosen because they represent those of other respondents and are well presented. The theme that emerged from the second interviews was subsequently classified into two sub-themes, namely support mechanisms from ISPs and support mechanisms from business associations (refer Table 6).

### Intention to comply with security control

Intention to comply with security control relates to individual compliance with the security support mechanism made available for small-scale traders. This study made an effort to measure the support mechanism through a key question. The key question was guided by several related questions for better response.

#### Major theme: support mechanisms (SM)

The key question for this section was: What support mechanisms are available to small-scale African migrant traders?

##### Sub-theme: support mechanisms from ISPs

This section discusses the findings on the various support mechanisms available to small-scale African migrant traders in Southern Africa from their ISPs. The following are some of the responses from the participants:

Most of the Lesotho traders are unaware of support mechanisms that they can get from ISPs. (Participant 19).

Sadly, the majority of Cameroonian traders do not take advantage of opportunities from ISPs and therefore they lack the necessary knowledge they need to protect their businesses from cyberattacks and other risks associated with online business transactions. (Participant 20).

Mozambican traders needs to familiarise themselves with the services provided by ISPs as far as cybersecurity awareness interventions are concerned so that they can take advantage of these free resources and use the information to protect their businesses from any cyber-related threats. (Participant 21).

##### Sub-theme: support mechanisms from business associations

This section presents the findings on the support mechanisms available to traders from their own business associations in the region. The following are some of the responses:

As the Somalian network we encourage our traders to take advantage of our monthly meetings so that they can learn from each other as to how they can protect their business from cyberattacks whilst transacting online. (Participant 16).

As the Kenyan business network, we encourage traders to remain vigilant while transacting online and [to] take advantage of the information resources we provide regularly so they can inform themselves about risks associated with digital transactions. (Participant 18).

As a Nigerian business network, we provide financial support through our stokvel to those traders in need whilst at the same time-sharing information in our monthly meetings where we talk about digital business, and the risks associated with online transactions as well as mitigation strategies so that their businesses are protected. (Participant 17).

From the findings above, it implies that the small-scale traders do not access necessary cyber awareness knowledge (refer SM, Participants 19, 20 and 21) that can protect their businesses from cyber-attack and associated cyber risks (refer SM, Participants 16, 17 and 18). According to Banga et al. (2021), exposure to cyber threats and risks has increased exponentially due to increased use of digital platforms for transactions, resulting in the loss of income and data privacy (Reva 2020). It is therefore of critical importance to protect devices against possible cyber threats that can negatively impact online trading. In the context of this study conceptual framework, the ability of the traders to be able to examine the security threats and implementing security policies represents “intention to comply with security control”.

## Discussion and research contribution

The study’s findings show that small-scale African migrant traders in Southern African are negligent due to limited knowledge of cyber-related threats. The information about cyber-related threats is available from various information portals such as the US-CERT Current Activity and the government department responsible for ICT. However, small-scale traders are unaware of the availability of this cyber-related information and therefore the limited knowledge about threats exposes them to hackers and other cybersecurity criminals because they are easier targets. It is recommended that small-scale traders have adequate security measures for their electronic computing devices that will prevent them from being exposed to cybersecurity threats. According to Matthew (2020), more than 90% of data breaches are due to negligence that might have been prevented with cybersecurity training, processes, procedures, and tools. Breitinger et al. (2020) claims that due to inadequate education, users tend to make poor security choices which increases the possibility of cybersecurity incidences. Hence, it is important to educate users about the risks associated with cybersecurity threats and to encourage them to use strong passwords and multiple authentication.

Additionally, the results of the study exhibit evidence of unprotected networks and uncontrolled access by third parties. Unsecured public networks, either at an airport, hotel, or coffee shop, are often accessible by anyone. Such networks often appeal to small-scale traders because of their free accessibility, which costs nothing in terms of data. Unauthorised access attacks can also occur in a network when the information systems networks are unprotected (Zaripova 2021). Attackers can exploit vulnerabilities in software, applications or systems to gain unauthorised access. However, the traders are unaware of the dangers to which such access exposes their trading activities.

Moreover, the small-scale African migrant traders are unable to protect their trading platforms from cybersecurity threats. Small-scale traders rarely consider installing security software to protect their trading platforms from cyber-related security exposure. This inability could be as a result of lack of knowledge of installing the software or the cost of the software. Hence it exposes their trading platforms to intruders that can disrupt their business processes. In addition, the use of mobile phones as a primary method of trading has gained traction, especially during the COVID-19 pandemic, which compelled people to embrace digital means of transacting. However, not every mobile phone is able to transact digitally in an effective manner. Moreover, some of these mobile phones are susceptible to cyber threats due to their system configurations, making them easier targets for cyberattacks. As a result, traders who cannot afford higher-level sophisticated mobile phones whose design is more difficult for cybercriminals to access remain under constant fear of losing their revenues via online transactions and sensitive customer information stored in their mobile phones.

Furthermore, the results showed that ISPs could assist small-scale traders by providing cybersecurity awareness interventions, such as installing security software in platforms used for trading purposes to protect them from external cyber threats that could compromise their businesses. In this case, the public–private partnership would be beneficial in supporting small-scale traders with accessible and affordable systems security software that could help protect their online trading platforms. This is a policy imperative that can support better migration processes in the light of cross-border entrepreneurial activities.

## Conclusion

This study has shown that African migrant traders in Southern African have been exposed to cybersecurity threats due to transacting on digital platforms especially during the COVID-19 pandemic. Most of these traders are not adequately protected against online-related risks and threats. Cybercrime has increased in the COVID-19 era as trading online increased. This study recommends a multi-pronged approach involving the traders themselves to mitigate cybersecurity threats. Their locally based migrant business associations, ISPs, governments, and business chambers need to collaborate to create multiple support mechanisms to assist traders in coping with the changing economic and digital trading environment in the diaspora.

It is important to note that the Internet is the new social interaction platform and exacerbates susceptibility to harmful opportunities especially following the COVID-19 lockdown. Hence, there should be appropriate regulations aimed at protecting mobile devices used by small-scale migrant traders. The regulation should be worded in a such a way that the small-scale traders can easily recognise, avoid and report internet-related scams and other forms of cybercrime.

The outcome of this study suggests that targeted interventions are needed to safeguard internet-related entrepreneurship from cyber threats. Considering that the small-scale African migrant traders may not be knowledgeable enough in ICT field with regards to cybersecurity and related threats (Wynn and Olayinka 2021), it is imperative that the following recommendations are adopted in securing their business transactions.

Multi-level access control: This study recommends multi-level access control based on the findings. Access should only be permitted with multi-level security clearances to specific third parties (creditors and debtors) for trading purposes only (Aghili et al. 2022). While single access control is commonly adopted as a security measure, multi-level access control has proven to be more effective (Sarma et al. 2022).

Multiple authentication: Furthermore, this study proffers multiple authentication based on the findings. Every entry to the system must be subjected to multiple authentications to ensure that only positively identified and authenticated users gain access to the platform (Cho et al. 2020). While single authentication such as password authentication is a popular security measure, multiple authentication has been shown to offer superior security (Prabakaran and Ramachandran 2022).

Connections protection: This study also recommends connections’ protection based on the findings. Computer systems or mobile devices can use a Virtual Private Network (VPN) or a proxy to connect with an encrypted SSL connection (Ezra et al. 2022). This is important, especially if the devices are being used to connect to the Internet in public places and they contain confidential data. A VPN allows users to connect securely to another network over the Internet as most users already have end-to-end encryption with message authentication (Grechishnikov et al. 2019). With VPN, traders can access geo-restricted content and hide browsing activity on public Wi-Fi (Grechishnikov et al. 2019). In addition, ISPs need to be engaged to support small-scale African migrant traders, especially when they are transacting using digital platforms.

Virus/malware protection: In addition, this study suggests virus/malware protection based on the findings. Viruses are software programmes that are normally created by cybercriminals to infect vulnerable mobile devices and computers (Aslan et al. 2023). Other malware such as Trojans can access a mobile device by hiding in a programme as a screen saver (Fuentes 2020). They are destructive and can erase a device’s software programme (Aslan et al. 2023). In order to protect the owner’s information, mobile devices need to be protected by an anti-virus programme that is regularly updated.

Increase cybersecurity awareness among small-scale African migrant traders: Furthermore, this study recommends cybersecurity awareness for small-scale African migrant traders based on the findings. Small-scale traders’ business associations in Southern African should run relevant cybersecurity awareness programmes for their members. Traders are also encouraged to search online for information on how to protect themselves from cyberattacks (Trim and Lee 2019) and cybersecurity threats while transacting online (Vuță et al. 2022). A range of applications (Apps) continuously ask for permission to access data and functions they don’t need. Since the “terms and conditions” are often overwhelming, the majority of people do not read them and sometimes accept them without reading them, giving the owners of those Apps access to their private information (Trim and Lee 2019). They also expose their devices to malware that corrupts data though their mobile devices (Fuentes 2020). Moreover, cyber attackers gain illegal access to personal information which they can use to cause harm to owners of devices. Against this background, digital education is an important intervention for protection of mobile devices and their owners.

ISPs involvement: This study recommends ISPs involvement based on the findings. According to Sianturi and Ramli (2022), ISPs provide some security services to their customers which can be categorised into three different categories: fully external, full internal and partially internal/partially external. Fully external is a service which provides traders with security advice such as how to set up a firewall or offer customers free security products such as antivirus software (Killalea 2000; Buhaljoti 2019). Fully internal is a service with active ISP involvement in which they implement increased filtering to address any suspicious activity (Buhaljoti 2019). Partially internal/Partially external is a service in which the ISPs impose policies on traders to cause them to play a role in preventing unwanted traffic for example the ISP forces traders to approve emails received from unknown senders before an email is accepted (Sianturi and Ramli 2022).

While this study has provided some recommendations to safeguard small-scale African migrant traders from cyber threats; the study could have resulted with more effective security measures if the following aspects were also taken into considerations.

The study was limited to small-scale African migrant traders who operated their digital transactions via android operated mobile phones. Therefore, small-scale African migrant traders who operated on other gadgets such as laptops and iPad were excluded from the study. Moreover, due to time constraints, not all small-scale African migrants from all African countries were involved in the study. Therefore, the findings cannot be generalised to all small-scale traders from the rest of the continent. In addition, the sample size for this study is limited due to the qualitative method employed. Hence, future research should focus on collecting quantitative data with a larger sample size to provide insights on cybersecurity threats that small-scale African migrant traders in Southern Africa are exposed to as result of using digital platforms for trading.

Future research could investigate data privacy during public health emergencies such COVID-19 and how digital trading can be made safer for all traders, especially small-scale traders who often lack the technical capacity to protect their information systems from cybersecurity threats. Future research should focus on collecting quantitative data to provide insights on cybersecurity threats that small-scale African migrant traders in Southern Africa are exposed to as result of using digital platforms for trading.
